# Preparation and Properties of Boron Modified Phenolic Resin for Automotive Friction Materials

**DOI:** 10.3390/ma18071624

**Published:** 2025-04-02

**Authors:** Jianrong Liu, Jialin Guo, Juanli Deng, Shangwu Fan, Xide Cai, Sijie Kou, Shaobo Yang

**Affiliations:** 1 School of Materials Science and Engineering, Chang’an University, Xi’an 710064, China; 2024131008@chd.edu.cn; 2 Science and Technology on Thermostructural Composite Materials Laboratory, Northwestern Polytechnical University, Xi’an 710072, China; shangwu_fan@nwpu.edu.cn (S.F.); cxd1513701031@163.com (X.C.); kousijie@mail.nwpu.edu.cn (S.K.); shaobo_yang@mail.nwpu.edu.cn (S.Y.)

**Keywords:** automotive friction material, boron-modified phenolic resin, high heat resistance, friction and wear behavior

## Abstract

To address the thermal fade problem of brake pads, a boron-modified phenolic resin with better temperature resistance is intended to be developed. By introducing B-O bonds and high-temperature-resistant units, the thermal decomposition temperature of the phenolic resin will be increased. The modified resin is obtained through a step-growth polymerization reaction and then incorporated into the brake pad formulation to be hot-pressed into samples. The thermal decomposition temperature of the resin is measured by TGA, and the thermal fade performance of the brake pad samples is analyzed through friction and wear experiments. The results show that the introduction of B-O bonds and the doping of nano-alumina have increased the thermal decomposition temperature of the phenolic resin to 480 °C, meeting the expectation. Brake pads molded with this resin as an adhesive showed significantly better thermal degradation than those made with ordinary phenolic resin. Meanwhile, during the braking process, the brake pads made from this resin form a complete and continuous friction film, demonstrating good mechanical properties and thermal fade performance. The wear amount under the entire braking test is also acceptable. In addition, an exploration of the thermal fade mechanism is carried out.

## 1. Introduction

The automotive manufacturing industry constitutes an important part of the global manufacturing sector, with the demand for automobiles increasing annually. The braking system is one of the most vital components of a vehicle. Brakes primarily convert kinetic energy into heat energy and other forms of energy through the frictional action of the rubbing pair [1,2,3,4].

C/C-SiC composites have low density, corrosion resistance, and excellent thermal stability and mechanical properties. When used as brake discs, they exhibit outstanding friction and wear performance [5,6,7]. Since 2001, C/C-SiC brake discs have been gradually applied to high-performance sports cars of manufacturers such as Porsche and Ferrari. At present, the semi-metallic brake pads paired with them hold a large market share. Their main components include reinforcing fibers, fillers, and organic binders, with phenolic resin materials being the main type of organic binders [8,9].

Currently, resin-based friction materials are widely utilized due to their characteristics such as low specific gravity, low braking noise, minimal damage to the mating disc, ease of molding and processing, and low cost. The phenolic resin features high hardness, high compressive strength, strong corrosion resistance, good wettability to fillers, and good heat resistance, as well as low cost and simple production process, and has thus always been the preferred choice for the resin matrix in friction materials [10,11,12,13]. However, with the increase in the operating speed and load of modern mechanical equipment and vehicles, the friction materials prepared from ordinary phenolic resin can no longer meet the usage requirements. The molecular chain of ordinary phenolic resin contains a large number of phenolic hydroxyl groups and methylene groups, which are prone to thermal decomposition. During high-temperature or emergency braking, the friction materials are liable to experience thermal fade, subsequently leading to a decrease in the braking force of the automobile and an increase in the wear amount, which significantly limits their application potential [14,15,16,17].

Consequently, enhancing the thermal decomposition temperature of the matrix is of great significance for improving the performance of resin-based friction materials. To address this issue, this paper will mainly focus on boron-modified phenolic resin and select three modification methods: (1) Introduction of B-O bonds via boric acid and doping with alumina nanoparticles; (2) Introduction of B-O bond and rigid benzene ring via phenylboronic acid and doping with nano-alumina at the same time; (3) Introduction of B-0 bond via boric acid and introduction of furan ring via furfural. Among them, Benzene ring can withstand higher temperatures. Furfural possesses two functional groups, namely the furan ring (conjugated double bonds: C=C-C=C) and the aldehyde group (C=O), and exhibits high reactivity and good heat resistance [18]. γ-nano-alumina has a uniform particle size distribution, high purity, good dispersion, a large specific surface area, and is inert to high temperatures.

Semi-metal resin-based friction material samples are prepared by hot-pressing, and the friction and wear performance of the samples are tested using a scaled-down inertia test bench for automotive brakes. The aim is to compare and obtain the optimal phenolic resin modification scheme. Additionally, the surface morphology of the brake pads is observed using a scanning electron microscope, and the thermal fade theory is further analyzed in combination with chemical reactions.

## 2. Experiments

### 2.1. Materials

Phenol, 37% formaldehyde solution, furfural, sodium hydroxide (NaOH, granular), hexamethylenetetetramine, all analytically pure, supplied by Tianjin Damao Chemical Reagent Factory (Tianjin, China). Boric acid, phenylboronic acid, and γ-nano-alumina (γ-nano-Al_2_O_3_) were analytically pure and purchased from Shanghai Yien Chemical Technology Co., Ltd., Shanghai, China. All the above chemical reagents were used as received.

The friction materials were composed of steel fibers (average length 1.6 mm, average diameter 0.2 mm, Dezhou Fangyuan Steel Wool Fiber Co., Ltd., Dezhou, China) as the reinforcement and other components, as shown in Table 1.

### 2.2. Sample Preparation

#### 2.2.1. Preparation of Nano-Al_2_O_3_/BPR

In the mole ratio, phenol-formaldehyde-boronic acid = 1:1.2:0.2. Molten phenol, formaldehyde solution, and NaOH were added into a three-necked flask equipped with a spherical condenser tube and a mechanical stirring device [19]. The solution was stirred and heated to 65 °C~70 °C and held at that temperature for 1.5 h. In the second step, boric acid was added to this solution. The solution was heated to 105 °C~110 °C for 1 h. Thereafter, γ-nano-Al_2_O_3_ was added into the solution for a reaction of 30 min. The solution was cooled to room temperature. And the yellow-brown doped nano-alumina boron-modified phenolic resin mixed solution (nano-Al_2_O_3_/BPR) was obtained. Then, the mixed solution was put into a vacuum oven at 65 °C to dry for 24 h to remove excess water. Finally, the resin in the solid state is obtained. The samples doped with 1, 2, 3, 4, and 5 g of γ-nano-Al_2_O_3_ were named BPR-1, BPR-2, BPR-3, BPR-4 and BPR-5.

#### 2.2.2. Preparation of Nano-Al_2_O_3_/PBPR

It was stated that phenylboronic acid was required to be ten percent of the total mass of the phenolic resin. Phenol, formaldehyde solution, and NaOH were added into a three-necked flask equipped with a stirrer and a condenser. The solution was stirred and heated to 65 °C~70 °C and held at that temperature for 1.5 h. Beyond, phenylboronic acid is added to this solution. And the solution was heated to 130 °C~140 °C for 1 h [20]. Then, γ-nano-Al_2_O_3_ was added into the solution for a reaction of 30 min. The solution was cooled to room temperature. A dark reddish-brown mixed solution of boron-modified phenolic resin doped with nano-alumina (nano-Al_2_O_3_/PBPR) is then obtained. Finally, this solution was removed in a vacuum to remove the water, and the solid resin was obtained. The samples doped with 1, 2, 3, 4, and 5 g of γ-nano-Al_2_O_3_ were named PBPR-1, PBPR-2, PBPR-3, PBPR-4, and PBPR-5.

#### 2.2.3. Preparation of FPBPR

The formaldehyde-furfural solution (n_formaldehyde_:n_furfural_ = 1:0.1, 1:0.15, 1:0.2, 1:0.25, 1:0.3) was preformulated and loaded into a constant-pressure dropping funnel. Then, phenol and NaOH were added into a three-necked flask equipped with a stirrer, a condenser, and a funnel. The solution was warmed up to 70 °C~80 °C for 2.5 h of reaction. Thereafter, phenylboronic acid was added to this solution. The solution was stirred at 140 °C for 1.5 h. A black-brown solution of phenylboronic acid-modified phenolic resin with furfural as a cross-linking agent (FPBPR) was obtained at room temperature. Water in the resin solution was removed in a vacuum. Eventually, solid FPBPR was obtained. The samples doped with different masses of furfural were named FPBPR-1, FPBPR-2, FPBPR-3, FPBPR-4, and FPBPR-5.

#### 2.2.4. Preparation of Friction Materials

The solid resin sample was ground to a fine powder by a mortar and pestle to be used as an adhesive and was mixed with other components in a small Y-mixer (100 r/min). The homogeneously mixed powder was poured into a mold and hot pressed in a flatbed vulcanizing machine. During hot pressing, the final temperature of the vulcanizing machine was set to 180 °C. The vulcanizing pressure was 43 MPa and the holding time was 20 min. Besides, three to five exhausts were carried out during the warming period. The square material obtained by hot pressing was cut in half into a pair of friction discs. The length was 40 mm. The width was 20 mm. The height was 10 mm. The pair of friction plates were heat-treated in an oven at 210 °C and held at that temperature for 24 h. Thus, the heat-treated samples were used for the following characterization. Samples of brake pads thermoformed with phenolic resin, BPR-2, PBPR-3, and FPBPR-4 as bonding agents were named Pad pr, Pad A, Pad B, and Pad C, respectively.

### 2.3. Testing and Characterization

#### 2.3.1. Testing Methods

The bulk density and open porosity of the friction materials were measured by the Archimedes method. An analytical balance (AG135, Mettler Toledo, Zurich, Switzerland) with an accuracy of 0.0001 g was used to weigh the samples. The bulk density and open porosity were calculated by using Equations (1) and (2) as follows:

(1) $$\rho_{a}=\frac{m_{1}\rho_{H_{2}O}}{m_{3}-m_{2}}\times 100\%$$

(2) $$P_{b}=\frac{m_{3}-m_{1}}{m_{3}-m_{2}}\times 100\%$$

where *ρ_a_*, *P_b_*, *m*_1_, *m*_2_, and *m*_3_, measured bulk density (g/cm^3^), open porosity (100%), dry weight (g), weightlessness (g), and wet weight (g), respectively.

Brinell hardness was chosen in this study to characterize the macro-hardness of the friction material. The use of Rockwell hardness testers (HR-150A, Mage, Laizhou, China) was based on test method ISO 6506-1:2014. The size of the sample was 40 mm × 20 mm × 10 mm. The indenter diameter was selected as 10 mm. The test force was set as 9804 N for fiction materials and the duration time of the test force was set as 30 s. Thus, Brinell hardness was calculated by using Equation (3) as follows:

(3) $$HBW=0.102\times \frac{2F}{\pi D\left(D-\sqrt{D^{2}-d^{2}}\right)}$$

where *HBW*, *F*, *D*, and *d*, measured Brinell hardness, payload pressure (N), indenter diameter (mm), and indentation diameter (mm), respectively.

An electronic universal testing machine (ETM series, Wance, Shenzhen, China) was selected to measure the layer shear strength of friction materials. Based on the test standard GB/T-26739-2011, the size of the sample is 20 mm × 20 mm × 10 mm and the loading rate is 5 mm/min. The shear strength was calculated by using Equation (4) as follows:

(4) $$\tau =\frac{F}{A}$$

where *τ*, *F*, and *A*, measured shear strength (MPa), maximum shear force (N), and sheared area (mm^2^), respectively.

**Figure 1:**
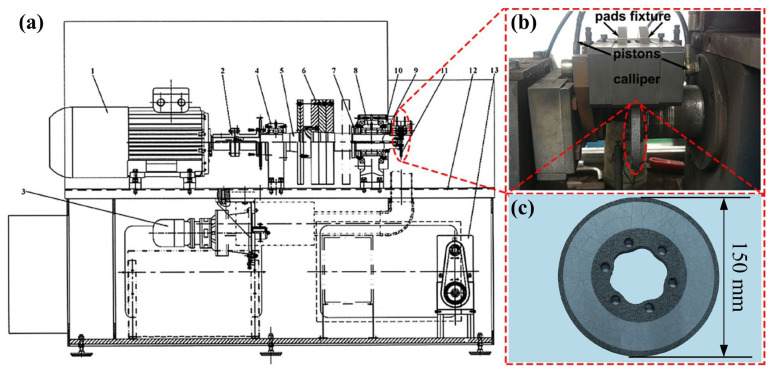
Schematic diagram of the test bench and physical drawing of brake disc. (**a**) schematic diagram [21]; (**b**) actual brake section [21]; (**c**) brake disc.

To simulate the real braking scenarios of automobiles, a scaled-down inertia test rig for automotive brakes (JF-122, Jilin Electrical and Mechanical Equipment Co., Ltd., Changchun, China) was used to conduct braking test experiments. As shown in Figure 1, Figure 1a is the whole test bench setup, Figure 1b is the brake component part of it, and Figure 1c is a carbon-ceramic disc with its surface coated with Si-SiC.

The brake pad size of the braking part is 40 mm × 20 mm × 10 mm, and the size of the brake disc is 150 mm in diameter and 10 mm in thickness. During the braking test, the surface temperature of the brake disc is measured using a contact-type K thermocouple. Next, the braking test program was determined by SAE-J2522 and detailed test conditions are summarized in Table 2. The frictional coefficient (COF) was calculated by using Equation (5) as follows:

(5) $$\mu =\frac{M}{2\times Arz\times R_{m}\times \eta \times P}$$

where *μ*, *M*, *Arz*, *Rm*, *η*, and *P*, measured COF, breaking torque (N·m), brake cylinder piston area (m^2^), brake lever arm (m), braking efficiency (100%), braking strength (kPa). At the end of the braking test, the thickness of the brake discs and pads was measured using a micrometer with an accuracy of 0.001 mm. The brake disc was measured at 12 points and then averaged. Brake pads were measured at 5 points and then averaged.

#### 2.3.2. Characterization

The structural composition of the modified resin was characterized by fourier transform infrared spectroscopy (FT-IR, TENSOR II, Bruker, Wurzbach, Germany). Prior to analysis, all samples were obtained by the compression method and transmission mode is used for testing. In this study, the thermal weight loss of the modified resin was tested by a thermogravimetric analyzer (TGA, SDT650, TA, USA). The test program was set to increase the temperature of the sample from room temperature to 800 °C in an air atmosphere at a rate of 10 °C/min. DSC isothermal curves of modified resins were obtained by differential scanning calorimetry (DSC, DSC300C, DZ, Beijing, China). The samples were rapidly heated up to 150 °C/180 °C/210 °C in an air atmosphere at a rate of 50 °C/min and then held at that temperature for half an hour. After braking tests, the surface morphology of brake discs and pads was characterized by an optical microscope (OM, YM710TR, Prague, Czech Republic) and tungsten filament scanning electron microscope (SEM, Vega4, Tescan, Brno, Czech Republic), respectively.

## 3. Results and Discussion

### 3.1. Properties and Characterization of Resins

#### 3.1.1. Structure of Modified Phenolic Resins

Due to the relationship between the infrared characteristic peaks of nano-sized aluminum oxide and its specific surface area and dehydration state [22,23], the characteristic peaks are complex. Since γ-nano-Al_2_O_3_ was only physically blended into the phenolic resin in this experiment, the infrared characteristic peaks of nano-alumina are not analyzed.

**Figure 2:**
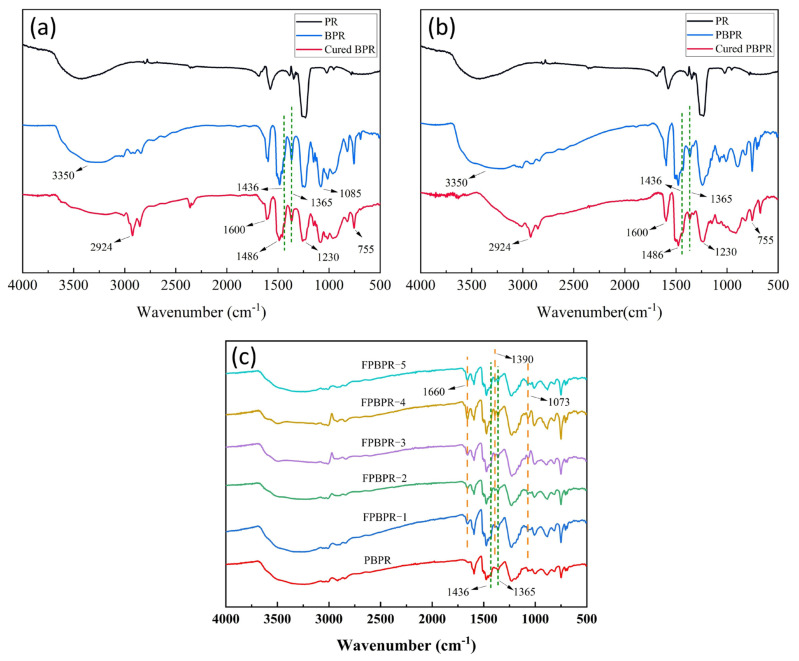
FTIR spectra of modified phenolic resins (**a**) BPR; (**b**) PBPR; (**c**) FPBPR.

Figure 2a shows the FTIR spectra of PR, BPR, and cured BPR. Figure 2b shows the FTIR spectra of PR, PBPR, and cured PBPR. The main absorption peaks are assigned as follows: 3350 cm^−1^ (C-OH), 2924 cm^−1^, 1085 cm^−1^, 755 cm^−1^ (C-H), 1600 cm^−1^, 1486 cm^−1^ (C=C, benzene ring), 1436 cm^−1^ (B-O, coordinate bond), 1365 cm^−1^ (B-O), and 1230 cm^−1^ (C-O). Notably, the peak intensities at 1085 cm^−1^ and 755 cm^−1^ (C-H stretching vibrations of the benzene ring) and at 1600 cm^−1^ and 1486 cm^−1^ (C-C stretching vibrations of the benzene ring) remain unchanged.

In comparison with PR, new absorption peaks appear at 1436 cm^−1^ and 1365 cm^−1^ in the spectra of BPR and PBPR, indicating the presence of B-O in the molecular structure. Additionally, the peak intensities at 3350 cm^−1^ (O-H stretching of phenolic hydroxyl) and 1230 cm^−1^ (C-O stretching of alcoholic hydroxyl) decrease. This reduction is due to the esterification reaction between B-OH in boric acid and some phenolic and alcoholic hydroxyl groups, forming borate esters or phenylboronic esters, which leads to a reduction in the content of phenolic and alcoholic hydroxyl groups.

Compared to BPR and PBPR, the peak intensities at 2924 cm^−1^ (C-H stretching vibrations of alkanes) in cured BPR and cured PBPR increase, indicating that during the curing process, some B-OH and phenolic hydroxyl groups continue to undergo dehydration condensation with alcoholic hydroxyl groups, leading to an increase in the number of methylene groups within the molecular chains. Therefore, it can be known that, regardless of whether the reaction is with boric acid or phenylboronic acid, B-O is ultimately introduced into the phenolic resin through esterification reactions, and the reaction becomes more complete after curing.

Figure 2c displays the FTIR spectra of PBPR and FPBPR with different furfural contents. Both PBPR and FPBPR show absorption peaks at 1436 cm^−1^ and 1365 cm^−1^, confirming the incorporation of B-O. Compared to PBPR, FPBPR exhibits absorption peaks at 1660 cm^−1^ (C=O stretching vibration of furfural) and 1390 cm^−1^ (C=C stretching vibration of furfural). The intensity of these peaks increases with higher furfural content, which can be attributed to the ortho or para addition reaction between the -CHO group on the furan ring and phenolate ions, incorporating the furan ring into the molecular chain. Furthermore, the intensity of the peak at 1073 cm^−1^ (C-O-C stretching vibration) also increases with higher furfural content. This results from the intermolecular dehydration between the alcoholic hydroxyl group on the furan ring and the phenolic hydroxyl group, forming more C-O-C bonds. This further demonstrates the incorporation of furfural into the molecular chain.

#### 3.1.2. Thermogravimetric Properties of Modified Phenolic Resins

**Figure 3:**
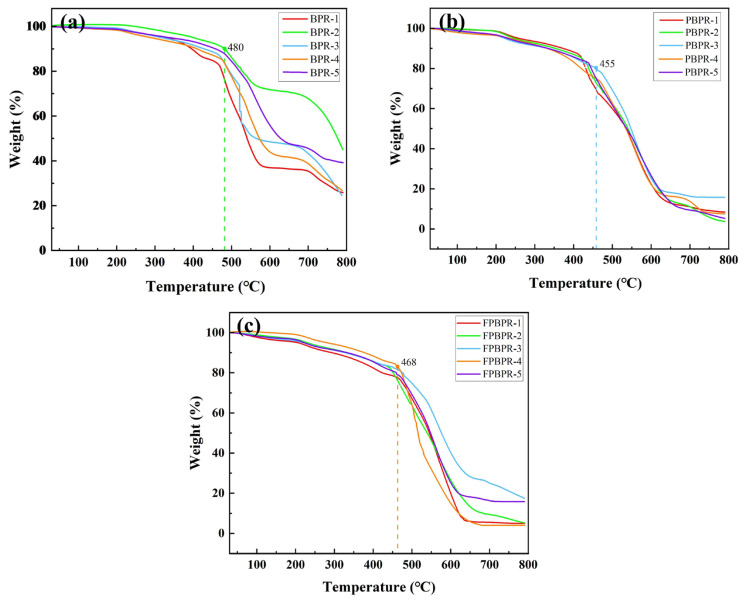
TG curves of weight loss for the modified phenolic resins. (**a**) BPR; (**b**) PBPR; (**c**) FPBPR.

The thermogravimetric analysis (TGA) of modified phenolic resins was conducted in an air atmosphere, and the thermogravimetric curves for the three types of modified phenolic resins are shown in Figure 3. Figure 3a presents the TGA curves for boric acid-modified phenolic resins doped with different contents of γ-nano-Al_2_O_3_. Generally, a noticeable decrease in weight occurs only after the temperature exceeds 450 °C, with the initial weight loss rate remaining below 15%. The carbon residue at 800 °C ranges from 25% to 45%, with some of this attributed to the presence of nano alumina. This behavior is due to the introduction of B-O bonds in the molecular chain of the phenolic resin, which possesses higher bond dissociation energies compared to C-C, C-O, and C-H bonds. The higher energy requirement for breaking B-O bonds makes them less prone to decomposition in high-temperature environments. Additionally, the incorporated γ-nano-Al_2_O_3_ exhibits high thermal stability. The synergistic effect of these factors leads to an increased thermal decomposition temperature for the modified phenolic resins compared to conventional phenolic resins (280 °C~350 °C).

Examining the individual samples, the thermal decomposition temperatures of BPR-2, BPR-3, BPR-4, and BPR-5 are all higher than that of BPR-1. Furthermore, the thermal decomposition temperatures of BPR-1, BPR-3, BPR-4, and BPR-5 exhibit a gradually increasing trend, indicating that the incorporation of γ-nano-Al_2_O_3_ enhances the thermal stability of the resin. The amount of γ-nano-Al_2_O_3_ doping is roughly proportional to the thermal decomposition temperature of the resin. Among these, BPR-2 demonstrates the best thermal stability, initiating thermal decomposition only at temperatures exceeding 480 °C. Its weight loss rate is 10%, and the residual carbon rate at 800 °C is 45%. This optimal performance may result from the ideal ratio of γ-nano-Al_2_O_3_, where steric hindrance effects are minimized, allowing for a uniform distribution within the resin’s molecular chains.

Figure 3b shows the TGA curves for phenylboronic acid-modified phenolic resins doped with varying amounts of γ-nano-Al_2_O_3_. A significant weight loss trend becomes evident after the temperature exceeds 415 °C, with weight loss rates remaining below 25%. The carbon residue at 800 °C ranges between 5% and 20%. The thermal decomposition temperatures of PBPR-2, PBPR-3, PBPR-4, and PBPR-5 are all higher than that of PBPR-1. Among these, the thermal decomposition temperatures of PBPR-1, PBPR-2, and PBPR-5 show a gradual increase, while the weight loss rate during decomposition exhibits an increasing trend. This suggests that both the introduction of B-O bonds and the incorporation of γ-nano-Al_2_O_3_ contribute to enhancing the thermal decomposition temperature. However, the method of introducing B-O bonds via phenylboronic acid, which contains a benzene ring, leads to significant steric hindrance. As the amount of γ-nano-Al_2_O_3_ increases, the effective participation of phenylboronic acid in the reaction diminishes, resulting in a reduced number of high-temperature-resistant B-O bonds and potentially leading to decreased thermal stability of the resin. Notably, PBPR-3 has the highest thermal decomposition temperature of 455 °C. Its weight loss rate is 20%, and the residual carbon rate at 800 °C is 20%. This may indicate a suitable balance between the introduction of B-O bonds and the doping of γ-nano-Al_2_O_3_.

Figure 3c illustrates the TGA curves for phenylboronic acid-modified phenolic resins crosslinked with different amounts of furfural. The thermal decomposition temperatures of these samples are relatively similar, ranging between 440 °C and 460 °C, except for FPBPR-2, which shows no significant differences compared to the others. As the furfural content increases, there are no discernible trends in either the weight loss rate or carbon residue. This lack of correlation may be attributed to the presence of a furan ring in furfural and a benzene ring in phenylboronic acid, both of which impose steric hindrance effects that limit their interaction. Consequently, the effective participation of both the benzene ring and B-O bonds, as well as the furan ring, is restricted, thus limiting the further enhancement of the resin’s thermal stability. Among these samples, FPBPR-4 exhibits the highest thermal decomposition temperature at 468 °C.

#### 3.1.3. Curing of Modified Phenolic Resins

**Figure 4:**
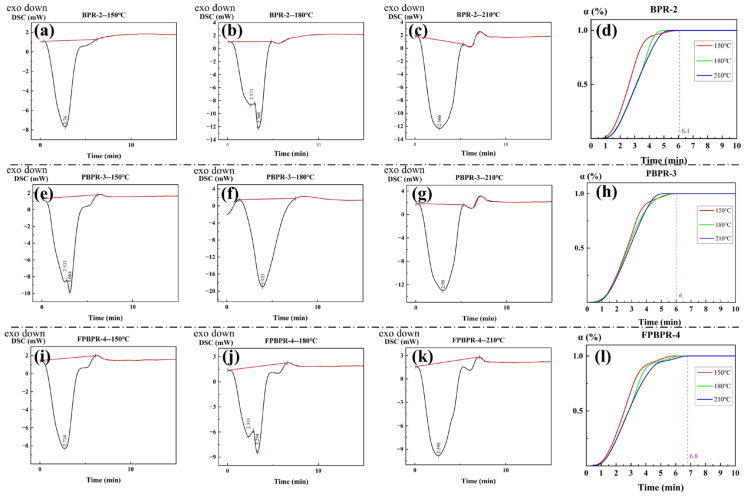
DSC isothermal curves and corresponding curability curves of modified phenolic resins. (**a**,**e**,**i**) 150 °C isothermal curve of BPR-2, PBPR-3, FPBPR-4; (**b**,**f**,**j**) 180 °C isothermal curve of BPR-2, PBPR-3, FPBPR-4; (**c**,**g**,**k**) 210 °C isothermal curve of BPR-2, PBPR-3, FPBPR-4; (**d**,**h**,**l**) Curing degree curve of BPR-2, PBPR-3, FPBPR-4.

Figure 4 presents the DSC isothermal curves of three types of modified phenolic resins at different temperatures, along with the corresponding degree of cure curves derived from the fittings. The samples BPR-2, PBPR-3, and FPBPR-4 were subjected to rapid heating at 150 °C, 180 °C, and 210 °C, respectively, maintaining each temperature for 30 min to obtain the DSC isothermal curves, as shown in Figure 4a–c,e–g,i–k. All curves exhibited a distinct exothermic peak within the first 10 min, attributed to the curing reaction of the resin upon reaching a specific temperature, releasing heat. Following this, the curing reaction concluded within 5 to 10 min, resulting in a plateau in the curves.

By performing baseline fitting, integrating the areas, and calculating the area ratios, the degree of cure curves for BPR-2, PBPR-3, and FPBPR-4 at various temperatures were ultimately fitted, as depicted in Figure 4d,h,l. Overall, the curing process was completed within 10 min for all samples. Although the curing rate slightly decreased with increasing temperature, the time required for complete curing correspondingly reduced. This information can be utilized to design the curing process for the resin. However, due to the small sample size used in the experimental tests, which may slightly differ from actual conditions, appropriate adjustments to the curing time of the resin should be taken.

### 3.2. Characterization of Brake Pads

#### 3.2.1. Brinell Hardness and Shear Strength

**Figure 5:**
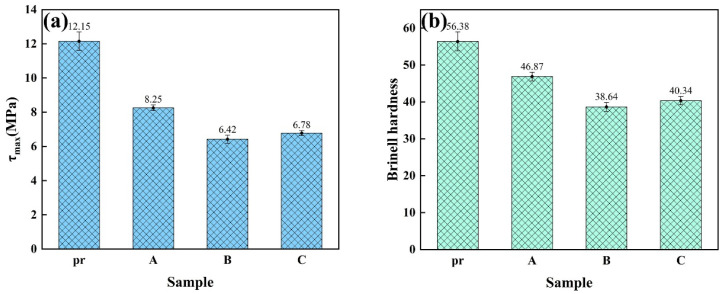
(**a**) shear strength of brake pads; (**b**) Brinell hardness of brake pads.

Shear strength and the Brinell hardness of the brake pads made from phenolic resin and three types of boron-modified phenolic resins are shown in Figure 5a,b. Shear strength and Brinell hardness of the Pad pr are the largest, indicating good bonding properties. However, too strong bonding properties lead to an increase in the denseness of the sheet, which results in an increase in the coefficient of friction. Therefore, the other three pads may be the most suitable in meeting the requirements of brake pads. The Brinell hardness of Pad A is 46.87, and the unit shear stress is 8.25 MPa. Whether it is the Brinell hardness or the shear strength, the values of Pad A are higher than those of Pad B and Pad C. And they meet the national standards, which shows that Pad A is better than Pad B and Pad C in mechanical properties. This has a positive effect on Pad A in the friction and wear tests. This is mainly because the resin used in Pad A has better bonding strength.

It can be seen from Table 3 that the Pad pr has the highest density. This also corresponds to the fact that the shear strength and Brinell hardness of the Pad pr in picture 5 are the largest. As can be seen in Table 3, the higher the open porosity, the lower the density of the sheet, which means that there are more pores on the surface and inside the pad. When the sheet is subjected to shear force, the stress cannot be well dispersed, resulting in localized stress concentration, which makes the pad more susceptible to damage and the shear strength decreases. After excluding its effect on the frictional coefficient, the density of Pad A is greater than that of Pad B and Pad C. Meanwhile, the open porosity of Pad A is smaller than that of Pad and Pad C.

#### 3.2.2. Friction and Wear Properties

**Figure 6:**
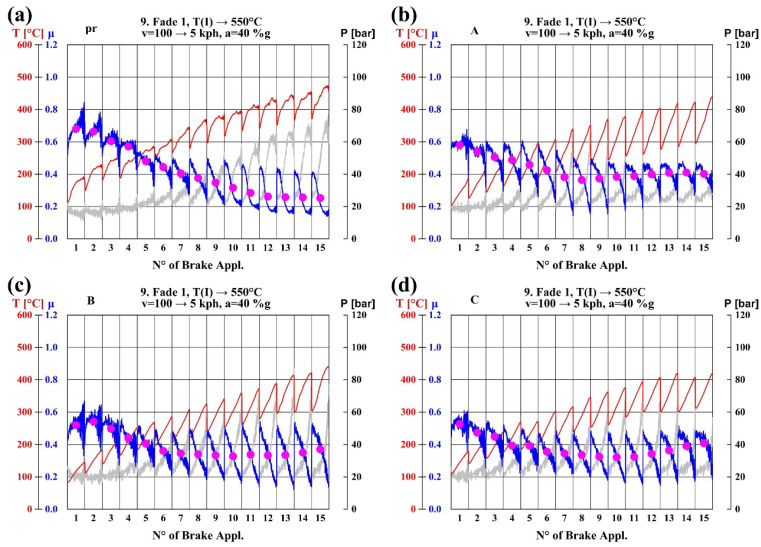
(**a**) The fade curve of Pad pr; (**b**) The fade curve of Pad A; (**c**) The fade curve of Pad B; (**d**) The fade curve of Pad C. (Grey line correspondent to P [bar]).

The thermal fade curves of the four types of pads are presented in Figure 6. As can be seen from Figure 6a, the frictional coefficient of Pad pr continues to decrease with increasing temperature, and the thermal fading phenomenon is very obvious. From Figure 6b, it can be known that with the increase of temperature, the frictional coefficient of Pad A firstly decreases and then slowly recovers to above 0.4. From Figure 6c,d, the frictional coefficients of Pad B and Pad C gradually recover as the temperature increases after decreasing to a certain range.

Combined with the performance of the four types of brake pads in the thermal fade stage and the above test data, it indicates that boron-modified phenolic resins are successfully demonstrated on brake pads. In a word, boron-modified phenolic resins largely improve the deficiencies of ordinary phenolic resins in the thermal fading of brake pads.

**Figure 7:**
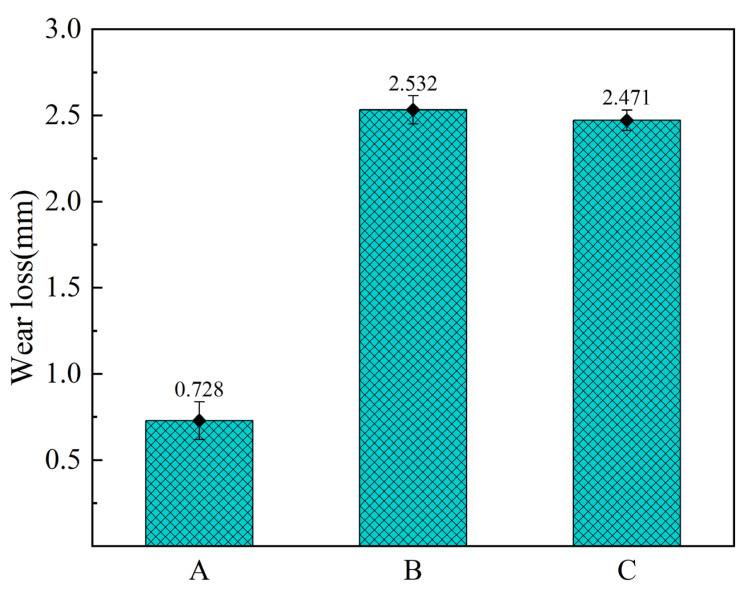
Histogram of brake pads wear loss.

Figure 7 illustrates the amount of wear loss for the three types of brake pads. From the Figure 7, it can be seen that Pad A has the smallest wear loss of 0.728 mm, while Pad B and Pad C have much larger wear loss than Pad A. The combination of thermal degradation and wear loss suggests that the selection of BPR-2 as the brake pad binder is the most suitable one.

### 3.3. Thermal Fade

To explore the causes of the thermal fade of the brake pads, the thermal fade braking process of the rubbing pair was divided into three stages:

(1) The first stage ends at the fifth braking, with a temperature of 350 °C, corresponding to the initial descending stage of the thermal fade curve.

(2) The second stage ends at the tenth braking, with a temperature of 420 °C, corresponding to the point where the thermal fade curve drops to its lowest level.

(3) The third stage ends at the fifteenth braking, with a temperature of 480 °C, corresponding to the recovery stage in the later period of the thermal fade curve.

#### 3.3.1. Surface Morphology of Brake Discs

**Figure 8:**
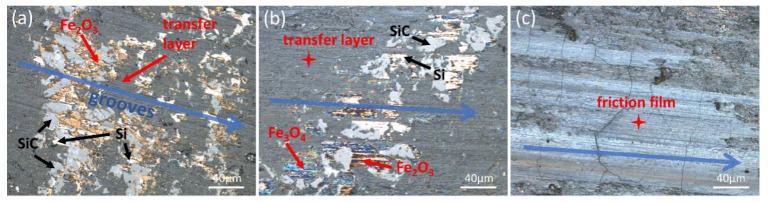
Surface morphology of brake disc at different stages of thermal fade. (**a**) Surface morphology of the first stage; (**b**) Surface morphology of the second stage; (**c**) Surface morphology of the third stage.

Figure 8 shows the surface morphologies of the brake disc at three stages. The surface of this disc is mainly composed of Si and SiC. In Figure 8a, the light gray area is composed of SiC, the bright white area is Si, and the golden yellow part is Fe_2_O_3_. Some areas on the surface of the brake disc are covered by a transfer layer formed by the wear debris of the friction material. The blue arrows indicate the scratches on the disc surface and the braking direction of the rubbing pair. In Figure 8b, a new blue area appears, and the composition of this part is Fe_3_O_4_, and most areas on the surface of the brake disc have been covered by the transfer layer. In Figure 8c, the original appearance of the disc itself can hardly be seen but is instead replaced by a complete and continuous friction film.

From the first stage to the second stage, the transfer layer gradually covers the disc surface. However, compared with the process from the second stage to the third stage, the covering speed of the transfer layer is not very fast. This is because the temperature in the early stage of thermal fade is lower than that in the later stage. In the early stage, the wear debris of the friction material will transfer to the surface of the brake disc, and at the same time, the wear debris of the brake disc will also transfer to the surface of the friction material (adhesion mechanism). In the later stage of thermal fade, as the temperature rises, the transfer is mainly that of the wear debris of the friction material to the surface of the brake disc (diffusion mechanism) [24]. It can be inferred from this that the friction film was not fully formed in the early and middle stages, which led to the occurrence of thermal fade. In the later stage, a complete and continuous friction film was formed, improving the friction coefficient and making it stable.

#### 3.3.2. Surface Morphology of Brake Pads

**Figure 9:**
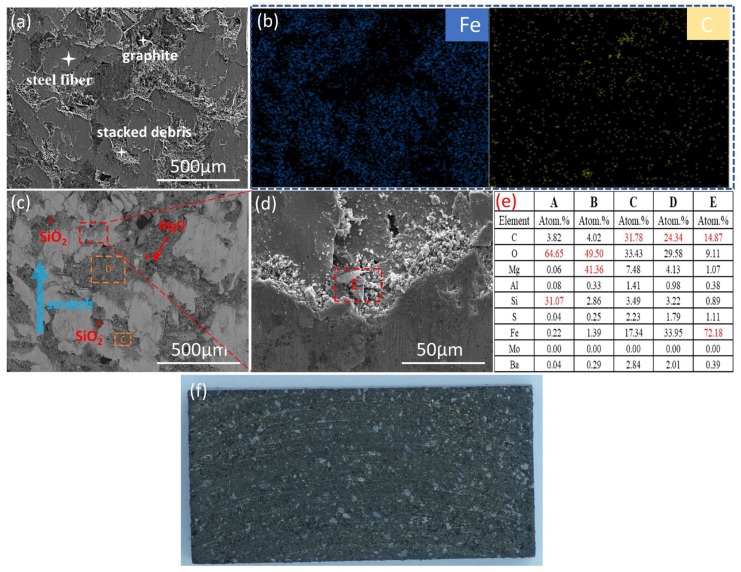
Surface morphology of brake pads at the first stage. (**a**,**d**) Secondary electron scans at different magnifications; (**c**) Backscattered electron scan; (**f**) Macroscopic surfaces; (**b**,**e**) EDS results.

The surface morphologies of the brake pads in the first stage of thermal fade are presented in Figure 9. In Figure 9a, according to face scanning results in picture (b), most areas form the main contact platforms composed of steel fibers, while a small number of areas accumulate the secondary contact platforms by the accumulation areas of graphite with larger particle sizes and wear debris. However, each contact platform is relatively independent and has a certain height difference. In Figure 9c, the larger particle phases that can be observed are known to be SiO_2_ and MgO respectively through EDS point scanning analysis of Figure 9e, because they have larger Mohs hardness and particle sizes. Analyzing Region C, the wear debris is mainly composed of graphite, steel fibers, oxides, and other formulation materials such as MgO and Al_2_O_3_. Analyzing Region D, its composition is similar to that of Region C, indicating that some steel fibers have friction materials adhered to their surfaces. When the red box area in Figure 9c is magnified, it can be seen that it is covered with a lot of debris. Through point scanning analysis, it is known that the debris is mainly composed of iron filings and a small amount of graphite, which also shows that during the process of the steel fibers extending outward, the shed iron filings and other debris particles continuously accumulate and form a secondary contact platform. From Figure 9f, it can be seen that there are slight scratches on the surface of the brake pad, accompanied by a yellow substance, which is presumed to be Fe_2_O_3_ [25,26], and it is possible that an oxide film formed on the surface of the brake pad.

**Figure 10:**
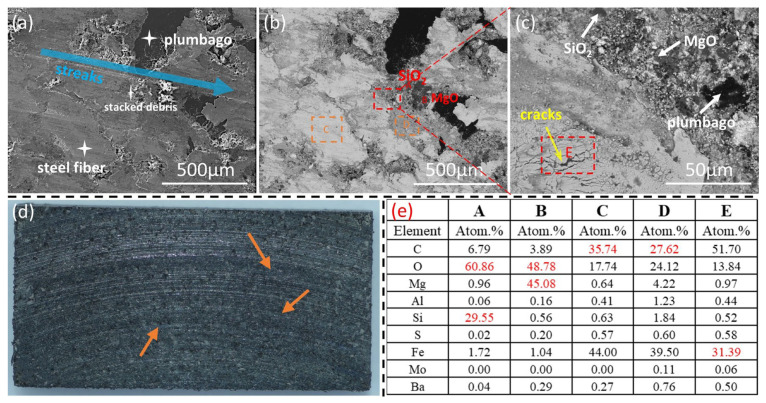
Surface morphology of brake pads at the second stage. (**a**) Secondary electron scan; (**b**,**c**) Backscattered electron scans at different magnifications; (**d**) Macroscopic surfaces; (**e**) EDS results.

Figure 10 shows the surface morphologies of the brake pads in the second stage of thermal fade. As shown in Figure 10a, the connection between the main contact platform composed of steel fibers and the secondary contact platform composed of wear debris becomes tighter, with no obvious height difference, and the whole look much smoother. Due to the high Mohs hardness of SiO_2_ and MgO, which are not prone to wear, the relatively obvious particle phases can still be seen in Figure 10b. The EDS point scanning results of Figure 10e indicate that the components of Region C and Region D are still mainly wear debris. Compared with the first stage of thermal fade, more areas on the steel fibers are adhered to by the friction materials and the amount of graphite increases. Figure 10c is the magnification of the red box area in Figure 10b. The wear debris accumulation area in the upper part is relatively flat, and the contact between the abrasive grains is close. In the wear debris accumulation area E, due to the insufficiently compact accumulation, cracks are formed after being subjected to stress. It can be presumed that these changes mentioned above have a positive impact on the further reduction of COF. Figure 10d shows that the scratches on the surface of the brake pad deepened. Upon closer inspection, it can also be found that a small amount of blue substance where the orange arrows point appeared on the surface of the brake pad, and it is presumed that a new oxide film started to form on its surface.

**Figure 11:**
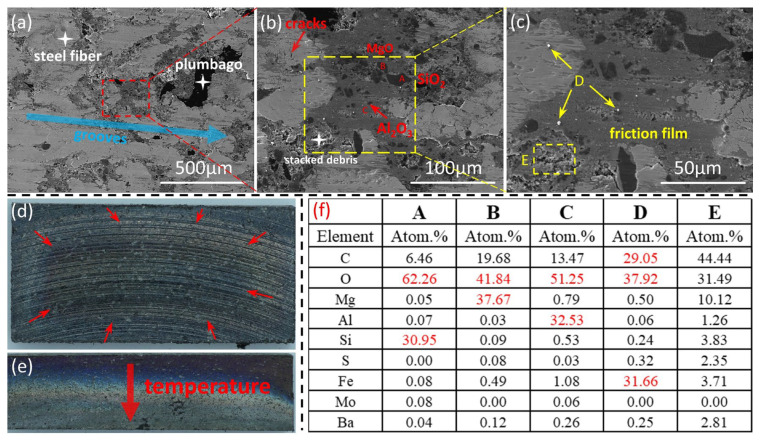
Surface morphology of brake pads at the third stage. (**a**) Secondary electron scan; (**b**,**c**) Backscattered electron scans at different magnifications; (**d**,**e**) Macroscopic surfaces; (**f**) EDS results.

Figure 11 shows the surface morphologies of the brake pads in the third stage of thermal fade. As shown in Figure 11a, the connections between the various contact platforms are so tight that it is almost impossible to distinguish their boundaries, and a complete and continuous friction film is formed. Through point scanning analysis, the friction materials are evenly dispersed on the surface of the brake pad. In Figure 11c, through EDS point scanning analysis of Figure 11f, Region E is an area of wear debris accumulation. It can be seen that the connections between the wear debris are getting closer, forming a smooth surface. In addition, there are many white shiny particles distributed on the friction interface. Analyzing their composition, they are relatively hard particles containing a large amount of iron filings. From this, it can be presumed that in the third stage of thermal fade, three-body abrasive wear mainly occurs between the friction interfaces, and obvious scratches can be seen in Figure 11d. In Figure 11d,e, large areas of blue substances appear on the surface of the brake pads. Judging from the temper colors, they are mainly Fe_3_O_4_. Along the direction of the red arrow, the temperature gradually rises, indicating that the temperature of the friction pads is mainly concentrated in the central area.

From the first stage to the third stage of thermal fade, due to the good ductility of steel fibers, they gradually spread out on the friction surface as the temperature rises. Under the action of stress, the particle size of the wear debris decreases and tightly accumulates to form a secondary contact platform. Some of the wear debris will adhere to the surface of the steel fibers while wrapped with a large amount of graphite, improving the lubrication. Then, the primary contact platform and the secondary contact platform jointly form a complete and continuous friction film. With the assistance of the surface oxide film, the coefficient of friction will recover to some extent until it stabilizes.

#### 3.3.3. Analysis of Thermal Fade

**Figure 12:**
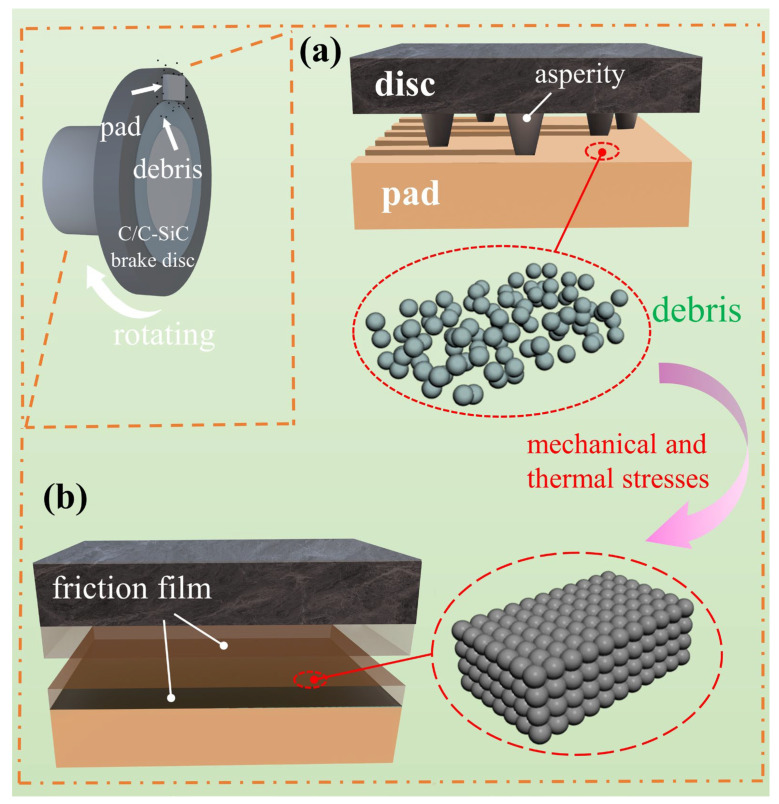
Modeling diagram for thermal fade.

The diagram of the thermal fade model is presented in Figure 12. Gray and black parts represent brake disc, orange parts represent brake pad. Figure 12a shows that in the pre-stage of thermal fade, the micro-convex body on the surface of the brake disc and the surface of the brake pad are undergoing a “plow effect”, which destroys the surface structure of the brake pad. Figure 12b shows the post stage of thermal fade, where a friction film is formed on the surface of both the brake pad and the brake disk. The specific mechanism is analyzed as follows. During the thermal fade stage, the temperature of the brake pads will increase with the increase in the number of braking operations.

In the preliminary stage, the displayed temperature does not reach the thermal decomposition temperature of the resin. In principle, the frictional coefficient should not drop significantly. However, an external thermocouple was used in this experiment, and the actual temperature at the contact surface between the brake disc and the brake pads is higher than the displayed temperature and may have already reached the thermal decomposition temperature of the resin. Consequently, the resin begins to decompose, the adhesion between components is lost, and the surface of the brake pads becomes loose and easily damaged by the hard asperities on the surface of the brake disc. Meanwhile, the frictional coefficient decreases accordingly.

In the post stage, the wear debris shed from the surface of the brake pads is transferred to the surface of the brake disc, resulting in adhesive wear. The frictional coefficient continues to decline. At the same time, as the temperature rises, the residual wear debris on the surface of the brake disc is transferred back to the surface of the brake pads [24]. Under the action of mechanical stress and thermal stress, the wear debris on the surface of the brake pads is gradually compacted, and the bonding relationship between the debris becomes close, forming a stable and flat friction film on the surface of the brake pads and disc, and the coefficient of friction is restored and stabilized.

## 4. Conclusions

To improve the thermal fade performance of the brake pads, this study started by increasing the thermal decomposition temperature of the phenolic resin. The means of introducing B-O bonds, rigid cyclic structures, and physically doping high-temperature-resistant components were adopted to modify the phenolic resin. The thermogravimetric results showed that the phenolic resin modified jointly by boric acid and γ-nano-Al_2_O_3_ had a higher thermal decomposition temperature, reaching about 480 °C. Moreover, the results of the friction and wear tests indicated that thermal fade performance of brake pads with boron-modified phenolic resin as binder is better than that of brake pads with ordinary phenolic resin as binder. This demonstrates that the improvement of the resin’s temperature resistance indeed has a positive effect on the thermal fade performance of the brake pads. Meanwhile, it also provides a basis for how to maintain the stability of the friction pad performance under higher braking temperatures in subsequent studies.

Furthermore, in the analysis of the causes of thermal fade, it was found that the reason for the decrease in the friction coefficient in the early stage is most likely still related to the thermal decomposition temperature of the resin. However, due to the lack of measurement of the actual temperature at the contact surface between the brake disc and the brake pads, a definite conclusion cannot be reached.

Currently, the thermal decomposition temperature of the resin studied in this experiment is still lower than 500 °C, and the problem of the temperature resistance of the resin has not been fundamentally solved. Nevertheless, this experiment verified that the increase in the thermal decomposition temperature of the resin has a positive impact on the thermal fade performance. This provides more ideas and solutions for the subsequent improvement of the friction and wear performance of the friction lining.

## Figures and Tables

**Table 1:** Friction material main formulations.

Components	Average Size (μm)	Mohs Hardness	Source
α-alumina	19	9	Henan Fengkai Refractories Co., Ltd. (Zhenzhou, China)
silicon dioxide	74	7	Heyuan Wanchuan Quartz Development Co., Ltd. (Heyuan, China)
graphite	250	2	Jiangxi Shuobang Material Technology Co., Ltd. (Fuzhou, China)
magnesia	44	5.5	Shanghai Ruhua Technology Co., Ltd. (Shanghai, China)
others	—	—	—

**Table 2:** Test conditions of SAE-J2522 [21].

Sections	Test Name	Conditions
1	Green μ characteristic	80→30 km/h; 3.0 MPa; 30 stops
2	Burnish	80→30 km/h; 1.5–5.1 MPa; 64 stops
3	Characteristic value 1	80→30 km/h; 3.0 MPa; 6 stops
4	Speed/pressure sensitivity	40→5 km/h; 1.0–5.0 MPa; 5 stops
5	—	80→40 km/h; 1.0–5.0 MPa; 5 stops
6	—	120→80 km/h; 1.0–5.0 MPa; 5 stops
7	—	160→130 km/h; 1.0–5.0 MPa; 5 stops
8	—	180→150 km/h; 1.0–5.0 MPa; 5 stops
9	Characteristic value 2	80→30 km/h; 3.0 MPa; 6 stops
10	Cold braking	40→5 km/h; 3.0 MPa; 1 stop
11	Motorway braking	100→5 km/h; 40% deceleration; 1 stop
		160→10 km/h; 40% deceleration; 1 stop
12	Characteristic value 3	80→30 km/h; 3.0 MPa; 18 stops
13	1st fading	100→5 km/h; 40% deceleration; 15 stops
14	Recovery 1	80→30 km/h; 3.0 MPa; 18 stops
15	Temperature/pressure sensitivity 100 °C	80→30 km/h; 1.0–5.0 MPa; 5 stops
16	Temperature/pressure sensitivity 500 °C	80→30 km/h; 1.0–5.0 MPa; 9 stops;
17		80→30 km/h; 1.0–5.0 MPa; 5 stops
18	Recovery 2	80→30 km/h; 3.0 MPa; 18 stops
19	2nd fading	100→5 km/h; 40% deceleration; 15 stops
20	Recovery 2	80→30 km/h; 3.0 MPa; 18 stops

**Table 3:** Density and open porosity of pads.

Simples	Density (g/cm^3^)	Open Porosity (%)
pr	3.83	9.88
A	3.54	10.76
B	3.35	10.98
C	3.47	10.84

## Data Availability

Data cannot be disclosed due to privacy.
